# Pre-pregnancy body mass index has greater influence on newborn weight and perinatal outcome than weight control during pregnancy in obese women

**DOI:** 10.1186/s13690-023-01025-2

**Published:** 2023-01-13

**Authors:** Jin Zhang, Rui Zhang, Jingjing Chi, Ya Li, Wenpei Bai

**Affiliations:** Department of Obstetrics and Gynaecology, Beijing Shijitan Hospital, Capital Medical University, Haidian, Beijing, 100038 China

**Keywords:** Body mass index (BMI), Gestational weight gain (GWG), Birth weight, Obesity, Pregnancy

## Abstract

**Background:**

The pre-pregnancy weight and gestational weight gain (GWG) are closely related to perinatal maternal and infant complications, but the relationship between pre-pregnancy weight and GWG and the pattern of interaction have not been reported. This study investigated the influence of weight control during pregnancy on the perinatal maternal and infant outcomes.

**Methods:**

A total of 835 singleton pregnant women who were hospitalized between January 2018 and December 2018 were retrospectively included in this study and divided into two groups: the diet guidance (DG) group (*n* = 167) and the control group (*n* = 668). The pre-pregnancy body mass index (BMI), GWG, and perinatal outcomes of the women and infants were determined in two groups.

**Results:**

The dietary modification and reasonable exercise during pregnancy effectively reduced the GWG, and even some women with pre-pregnancy obesity achieved weight loss during pregnancy. The GWG in the DG group was significantly lower than in the control group, especially in the second and third trimesters. GWG was positively related to birth weight. The birth weight in the DG group was significantly lower than in the control group when their mothers had similar GWG. In women with pre-pregnancy obesity, GWG seemed to be negatively related to birth weight. However, after adjusting the mean BMI, the pre-pregnancy BMI and GWG were positively related to the birth weight. Compared with the control group, the incidences of dystocia, postpartum hemorrhage, macrosomia, small for gestational age infants and neonatal complications significantly reduced in the DG group, and the preterm birth rate was comparable between two groups. Some women with pre-pregnancy obesity lose weight during pregnancy, but there was no premature birth or small for gestational age infant. The incidences of macrosomia, postpartum hemorrhage, dystocia, cesarean section and gestational diabetes increased significantly with the increase of pre-pregnancy BMI.

**Conclusion:**

For women with increased pre-pregnancy BMI, strict weight control is required to reduce obesity-related complications of the mother and infant. The weight control in the second and third trimesters is especially important and most likely to prevent GWG. Compared with GWG, pre-pregnancy BMI has greater influence on the birth weight and maternal and infant complications, and may even compromise the benefits of weight control during pregnancy. Thus, weight control is recommended before pregnancy.

## Background

The pre-pregnancy weight and gestational weight gain (GWG) are closely related to perinatal complications. Obesity as a consequence of social factors and hormones during pregnancy has become a common social phenomenon in pregnant women, and excessive increase in the GWG is common in clinical practice. Currently, obesity is one of the most critical health threats worldwide, and the incidence of obesity is still rising in women of childbearing age [1]. Pre-pregnancy obesity is associated with excessive GWG, gestational diabetes mellitus (GDM) [2], vaginal surgery-assisted delivery, cesarean section, and increased birth weight [3].

Pre-pregnancy obesity may cause spontaneous abortion and congenital embryo abnormality in early pregnancy [4], and the infants are also more likely to develop obesity in adulthood [5]. Moreover, pre-pregnancy obesity may increase the risk for metabolic diseases (such as insulin resistance) [6, 7] and have adverse effects on the neurocognitive development of infants [8–10]. Maternal obesity will affect the development of the fetal hypothalamus and the hypothalamic structure and function, resulting in fetal insulin resistance [11]. Moreover, maternal obesity will increase the risk of obesity-related cardiometabolic diseases [12, 13], liver disease [14], kidney disease [15], and nervous system dysfunction [16, 17] in infants.

In addition, excessive GWG may increase pelvic pressure, which aggravates the postpartum pelvic dysfunction during delivery, and affects postpartum recovery of body shape and the quality of life. Women with excessive GWG will develop body fat accumulation after delivery, and the elevation of GWG (according to the criteria of the Institute of Medicine [IOM]) may increase the risk of postpartum obesity [18], and also affect the pelvic muscle strength of women after delivery [17].

In 2009, the IOM divided the pre-pregnancy body mass index (BMI) as follows: underweight (BMI < 18.5 kg/m^2^), normal weight (BMI = 18.5–25 kg/m^2^), overweight (BMI = 25 ~ 30 kg/m^2^), and obesity (BMI = ≥30 kg/m^2^), and recommended GWG [19]. However, there is no individualized guidance for weight control in pregnant women, especially those with pre-pregnancy obesity. Therefore, it is necessary to develop a more suitable protocol for weight control during pregnancy, aiming to reduce maternal and infant complications. Our study retrospectively analyzed singleton pregnant women in our hospital, explored the characteristics of GWG in women with different pre-pregnancy BMI, and investigated the influence of weight control during pregnancy on the perinatal maternal and infant outcomes.

## Materials and methods

### Ethics statement

This study was approved by the Ethics Committee of the Beijing Shijitan Hospital of Capital Medical University (Beijing, China). Written informed consent was obtained from all subjects prior to study. All procedures were performed in accordance with the Declaration of Helsinki.

**Fig. 1:**
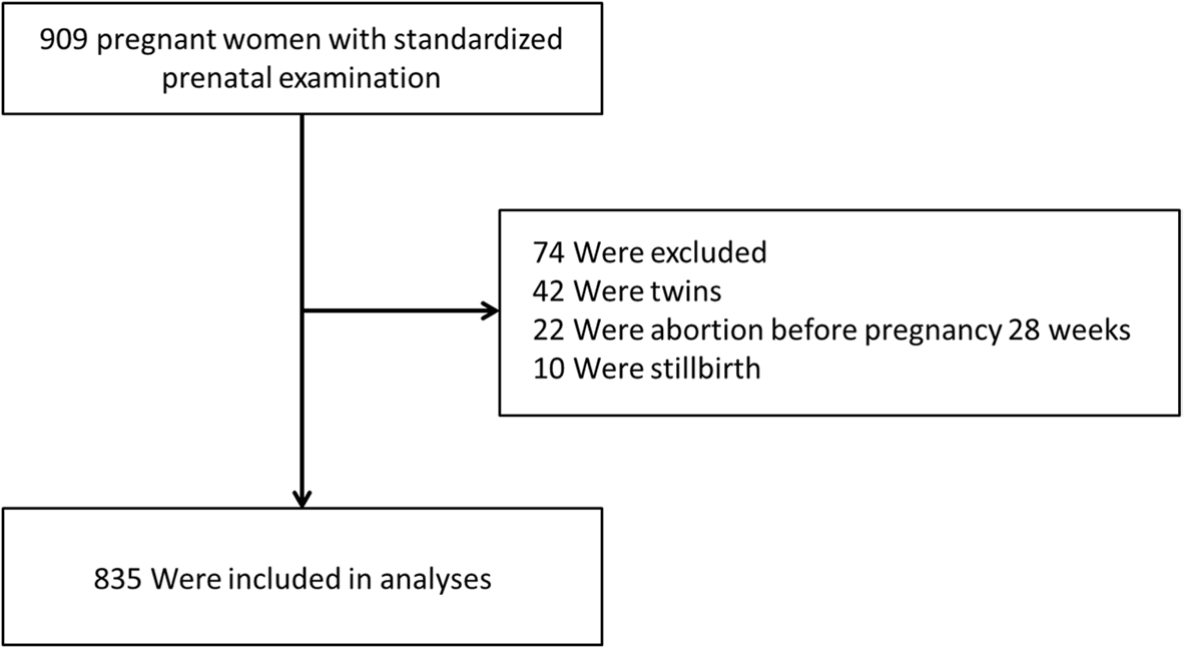
Inclusion of cohort. Singleton pregnant women were included in this study

Singleton pregnant women who were hospitalized in our hospital between January 2018 and December 2018 were included in the present study [19]. A total of 835 subjects met the inclusion criteria (Fig. 1). The mean age was 31.93 ± 3.84 years (range: 21–43 years); there were 357 primiparas and 478 multiparas (vaginal or surgical delivery). The exclusion criteria were as follows: women with bigeminal pregnancy, abortion before gestational 28 weeks or stillbirth.

#### Methods

All the pregnant women received routine prenatal examinations once every 4 weeks within the first 28 gestational weeks, once every 2 weeks between 28 and 36 gestational weeks, and once weekly after 37 gestational weeks. In the first prenatal examination, the body composition and diet were analyzed; the vital signs, body weight, medications, comorbidities, findings from physical and imaging examinations, clinical diagnosis, and medical prescriptions were also recorded in the following examinations.

Grouping: This was a retrospective study. All the pregnant women were divided into two groups: the diet guidance (DG) group and the control group. In the DG group, subjects received prenatal examinations and consulted obstetrics dietitians. In the control group, subjects received routine examinations without consultation with obstetrics dietitians.

In the DG group, there were 167 subjects. Each subject received guidance from an obstetric nutritionist according to the general condition and weight gain, the diet composition was adjusted, and the nutritional education and exercise guidance were administered: (1) At each prenatal examination, the pregnant women received education about the possible risks of excessive GWG (such as gestational diabetes mellitus and macrosomia), and the physicians emphasized the important of weight control during pregnancy, corrected their wrong knowledge about diet and asked them to cooperate with treatment to avoid excessive anxiety; (2) They were given low-glucose, low-carbon diet, and the coarse grains and dietary fiber increased in the diet; the daily energy intake did not exceed the recommended daily average energy (2000–2300 kcal/d for subjects with underweight, 1800–2100 kcal/d for subjects with normal weight, 1500–1800 kcal/d for subjects with overweight and obesity); (3) They were asked to exercise appropriately (10,000 steps/d in the second trimester and 20,000 steps/d in the third trimester) [20, 21]. Under the premise of normal blood pressure, breathing, blood glucose, and fetal heart rate, exercise 3 to 4 times daily with 30–40 min for each was recommended. The time interval between exercises was mainly based on the feeling of pregnant women, and strenuous exercise was avoided. The brisk walking or setting-up exercises for pregnant women were recommended with small weight and self-weight muscle training; the pregnant women were asked to monitor blood pressure and blood glucose before and after exercise, and then drink water 5–10 min after exercise. They were asked to record the information of daily exercises (such as steps, time of excises and time of resistance exercise) which was provided to the doctors at each examination; (4) Subjects received regular guidance via WeChat platform, and the data were recorded and summarized and then provided to the doctors at each examination.

In the control group, there were 668 subjects who received nutritional education once in the first trimester (6–12 weeks of gestational age), and they received oral diet guidance at each examination.

#### Observations

The GWG was defined as the difference between the self-reported pre-pregnancy body weight and the body weight before delivery. The GWG was recorded in three stages: the first trimester (difference between the self-reported pre-pregnancy body weight and body weight at 12 weeks of gestation age), the second trimester (difference between the body weight at 28 weeks and body weight at 12 weeks), and third trimester (difference between the body weight before delivery and body weight at 28 weeks). In addition, the cumulative weight gain was monitored at each stage.

The maternal outcomes included cesarean section, premature delivery (earlier than 37 weeks of gestation age at the time of delivery), dystocia (including assistant vaginal delivery and shoulder dystocia), postpartum hemorrhage (blood loss ≥500 ml in 24 h after vaginal delivery, or ≥ 1000 ml in 24 h after cesarean) [22] and GDM. Neonatal outcomes included macrosomia (birth weight ≥ 4000 g), small for gestational age (SGA, birth weight lower than the 10th percentile of the average weight at the same gestational age) [23], and neonatal complications (fetal distress and admission to neonatal intensive care unit [NICU]). The metabolic rate was calculated according to Cunningham equation [24]: BMR (cal/day) = 500 + 22 (LBM). Lean body mass (LBM) was calculated by individual nutrition analyzer NQA-Pplus (Beijing Sihai Huachen Technology Co., LTD).

#### Statistical analysis

The propensity score weighting was employed to adjust baseline variables, and the constructed weight variable effect estimation target was the average treatment effect (ATE), which was used to weight the outcome variable. Propensity score matching and propensity score regression were used for sensitivity analysis. The propensity score matching employed the greedy algorithm, and the ratio of subjects in the DG group to those in the control group was 1:3.

#### The outcomes were analyzed as follows

##### Influence of diet guidance on GWG

The cumulative GWG at different time points was used as the dependent variable, and the Mixed-Effect Model for Repeated Measures was used for analysis. The height, pre-pregnancy weight, group, time, and interaction between group and time were included in the model with a non-structural covariance structure. The Least Square (LS) mean of each group at different time points and its 95% CI, and the *P* value of intergroup LS mean were reported. P value of the main effects of the group was reported.

The GWG between two adjacent stages was used as the dependent variable, and the analysis of covariance model (ANCOVA) was used in which the height, pre-pregnancy weight, and group were included. The mean LS, 95% CI, and *P* value of groups at different stages were reported.

##### Influence of diet-guidance on the neonatal birth weight

Birth weight was used as the dependent variable, and the covariance analysis was employed for comparison between groups. The pre-pregnancy BMI, group, and interaction between the group and BMI were included in the model. If there was no significant difference in the interaction, it was removed from the model, and the *P* values of pre-pregnancy BMI and group were reported. If there was a significant difference in the interaction, only the results of the interaction were reported.

##### Influence of diet-guidance on the maternal and infant outcomes

Logistic regression analysis was used in which the height, pre-pregnancy weight, and group were included. The Odds Ratio (OR) of the group and its 95% CI were reported.

##### Influence of diet-guidance on the baseline metabolic rate in the first trimester

The baseline metabolic rate in the first trimester was used as the dependent variable, and ANCOVA was used in which the pre-pregnancy BMI, group and interaction between pre-pregnancy BMI and group were included. If there was no significant difference in the interaction, it was removed from the model, and the *P* values of pre-pregnancy BMI and group were reported. If there was a significant difference in the interaction, only the results of the interaction were reported.

##### Relationships of pre-pregnancy BMI and GWG with birth weight

Birth weight was used as the dependent variable, and the general linear model was employed for analysis. The pre-pregnancy BMI, GWG, and their interaction were included in this model. If there was no significant difference in the interaction, it was removed from the model, and the *P* values of pre-pregnancy BMI and GWG were reported. If there was a marked difference in the interaction, only the results of interaction were reported.

In this study, subjects were subdivided according to the pre-pregnancy BMI (< 18.5 kg/m^2^, 18.5–24.9 kg/m^2^, 25–29.9 kg/m^2^ and 30 kg/m^2^), and the consistency was employed among different subgroups. A value of *P* < 0.05 was considered statistically significant. SAS 9.4 was employed for statistical analysis.

## Results

**Fig. 2:**
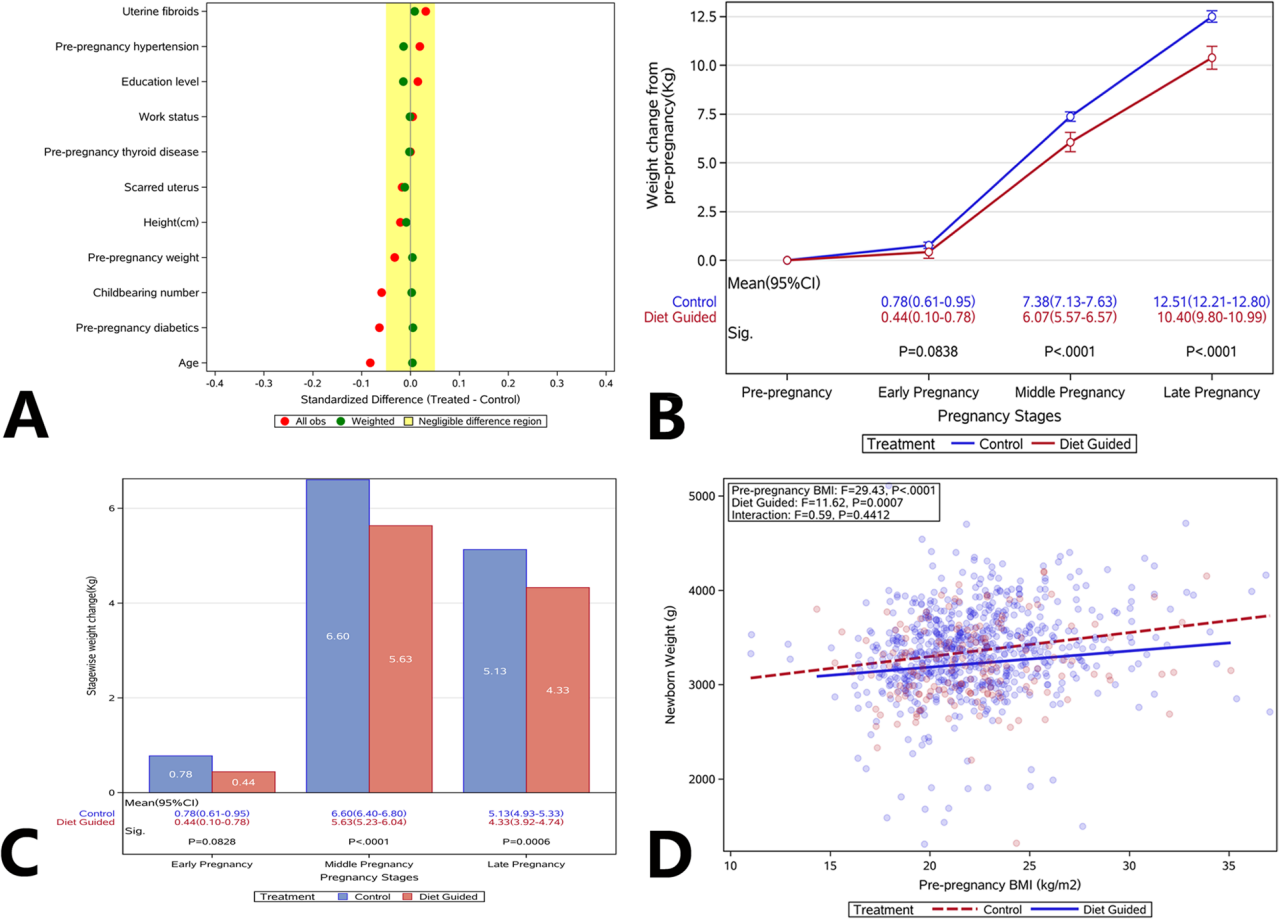
Comparison of clinical characteristics balance, gestational weight gain, and birth weight between DG group and control group. **A** The propensity score weighting was used to adjust the differences between groups and balance the parameters in two groups; **B** Comparison of cumulative GWG in the first, second and third trimesters. The rate of GWG in the DG group reduced, and significant difference was observed in the second and third trimesters between two groups. **C** Comparison of GWG in the first, second and third trimesters. The GWG in the DG group reduced significantly in the second and third trimesters; **D** Relationship of birth weight with pre-pregnancy BMI and diet guidance. The *P* values of pre-pregnancy BMI and diet guidance were all from the model after removal of their interaction (because there was no significant difference in the interaction). Body Mass Index (BMI); gestational weight gain (GWG); diet guidance (DG)

The age, height, pre-pregnancy weight, multipara, education level, working status, the status of scarred uterus, uterine fibroids, pre-pregnancy hypertension, pre-pregnancy thyroid disease, and pre-pregnancy diabetes were collected from the medical record. There were no marked differences in these parameters between the two groups (*P* > 0.05) (Table 1). In addition, propensity score weighting was used to adjust the differences between groups and balance the parameters in the two groups (Fig. 2A).

**Table 1:** Clinical characteristics of subjects in two groups

Characteristic	Diet guidance group	Control group	*P* value
Age —mean ± SD (yrs)	31.68 ± 3.80	32.00 ± 3.85	0.344
Height—mean ± SD (cm)	162.67 ± 4.93	162.77 ± 4.95	0.809
Pre-pregnancy weight —mean ± SD (Kg)	58.64 ± 10.66	58.97 ± 9.85	0.702
Multipara—no.(%)	93 (55.69%)	385 (57.63%)	0.356
High education—no.(%)	35 (20.96%)	136 (20.36%)	0.469
Jobless—no. (%)	21 (12.57%)	83 (12.43%)	0.523
Scarred uterus—no.(%)	24 (14.37%)	92 (13.77%)	0.463
Uterine fibroids—no.(%)	6 (3.59%)	28 (4.19%)	0.464
Pre-pregnancy hypertension—no.(%)	4 (2.40%)	18 (2.69%)	0.542
Pre-pregnancy thyroid disease—no.(%)	7 (4.19%)	28 (4.19%)	0.600
Pre-pregnancy diabetics—no.(%)	2 (1.20%)	4 (0.60%)	0.345

### GWG

As compared to the control group, the accumulative GWG reduced markedly in the DG group (Fig. 2B). The GWG was 0.44 kg (95%CI, 0.10–0.78) vs. 0.78 kg (95%CI,0.61–0.95) (*P* = 0.0838) in the first trimester, 6.07 kg (95%CI, 5.57–6.57) vs. 7.38 kg (95%CI, 7.13–7.63) (*P* < 0.0001) in the second trimester, and 10.40 kg (95%CI,9.80–10.99) vs. 12.51 kg (95%CI, 12.21–12.80) (*P* < 0.0001) in the third trimester. The F value of the main effect (diet guidance) was 29.91 (*P* < 0.0001), and the reduction was observed in each stage of pregnancy, especially in the second and third trimesters (*P* < 0.0001) (Fig. 2C).

### Maternal and infant outcomes in two groups

Overall, the GWG had a positive relationship with birth weight: the higher the GWG, the higher the birth weight was. As compared to the control group, the infants in the DG group were more likely to have lower birth weight when the GWG was similar between the two groups.

**Fig. 3:**
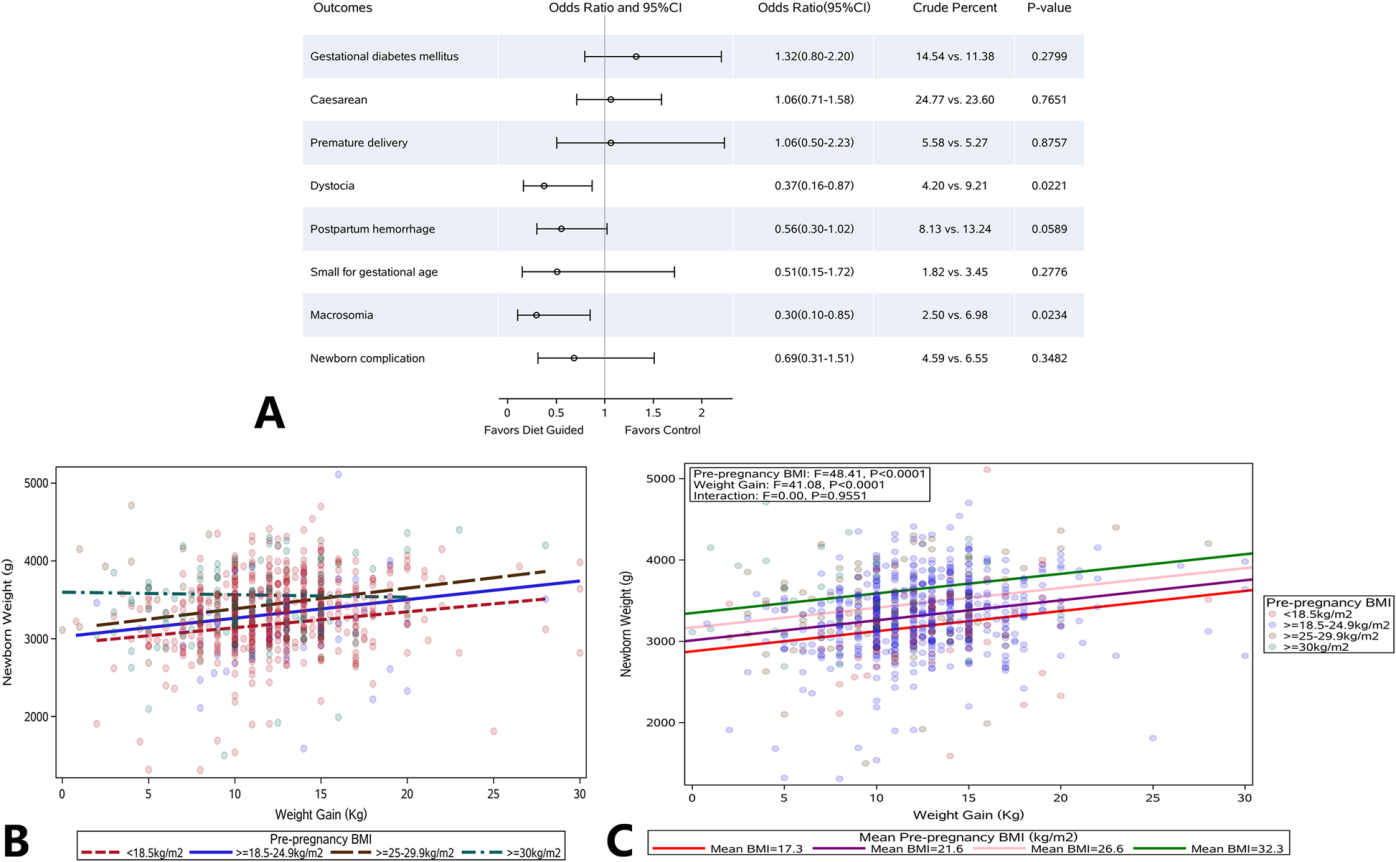
Comparison of perinatal outcomes of the women and infants between DG group and control group. **A** Comparison of clinical parameters between DG group and control group. The incidences of dystocia and macrosomia significantly reduced in the DG group (*P* < 0.05). The incidences of postpartum hemorrhage, SGA and neonatal complications significantly reduced in the DG group (OR < 1), but there were no significant differences in the incidences of GDM, cesarean section, and preterm birth between two groups. **B** The relationship between GWG and birth weight in pregnant women with different pre-pregnancy BMI (pre-pregnancy BMI was not adjusted); **C** The relationship between GWG and birth weight in pregnant women with different pre-pregnancy BMI (using mean BMI; after adjusting BMI before pregnancy). Body Mass Index (BMI); gestational weight gain (GWG); diet guidance (DG); gestational diabetes mellitus (GDM); small for gestational age (SGA)

Comparisons of clinical parameters: As compared to control group, the incidences of dystocia (4.20% vs 9.21%, *P* = 0.0221) and macrosomia (2.50% vs 6.98%, *P* = 0.0234) reduced significantly in the DG group. In addition, the incidences of postpartum hemorrhage, SGA, and neonatal complications reduced markedly in the DG group as compared to the control group (OR < 1). However, there were no significant differences in the incidences of GDM, cesarean section and premature delivery between two groups (Fig. 3A).

According to the pre-pregnancy BMI, the pregnant women were divided into underweight (BMI < 18.5 kg/m^2^), normal weight (18.5–25 kg/m^2^), overweight (25–30 kg/m^2^), and obesity (BMI ≥ 30 kg/m^2^) subgroups. In the four subgroups, the relationship between GWG and birth weight was evaluated. Results showed the GWG in the underweight, normal weight, and overweight subgroups were positively related to the birth weight: the higher the GWG, the higher the birth weight was. However, in the obesity subgroup, the GWG was negatively related to birth weight. After weight control, the birth weight increased with the reduction of GWG. It seemed that the GWG had no relationship with birth weight (Fig. 3B).

Thereafter, a model was employed to adjust the pre-pregnancy BMI in the four subgroups (Fig. 3C). As shown in Fig. 3C, the pre-pregnancy BMI and GWG had positive correlations with birth weight. If GWG was comparable, the higher the pre-pregnancy BMI, the higher the birth weight was. The large *P* value of the interaction indicates that the effects of pre-pregnancy BMI and GWG on the birth weight did not interact with each other, and independently affected the birth weight. The inverse relationship between GWG and birth weight in the obesity group might be ascribed to higher BMI. The pre-pregnancy weight still masked the effect of reduced GWG, although GWG was reduced in the obesity subgroup.

The fitted line in the figure was from the model: birth weight = a + b1 × pre-pregnancy BMI + b2 × GWG + b3 × pre-pregnancy BMI × GWG. The average pre-pregnancy BMI in four subgroups was included into the model, and four regression lines were obtained. The *P* value of pre-pregnancy BMI and GWG was from the model after removing the interaction between pre-pregnancy BMI and GWG (there was no significant difference in the interaction).

**Fig. 4:**
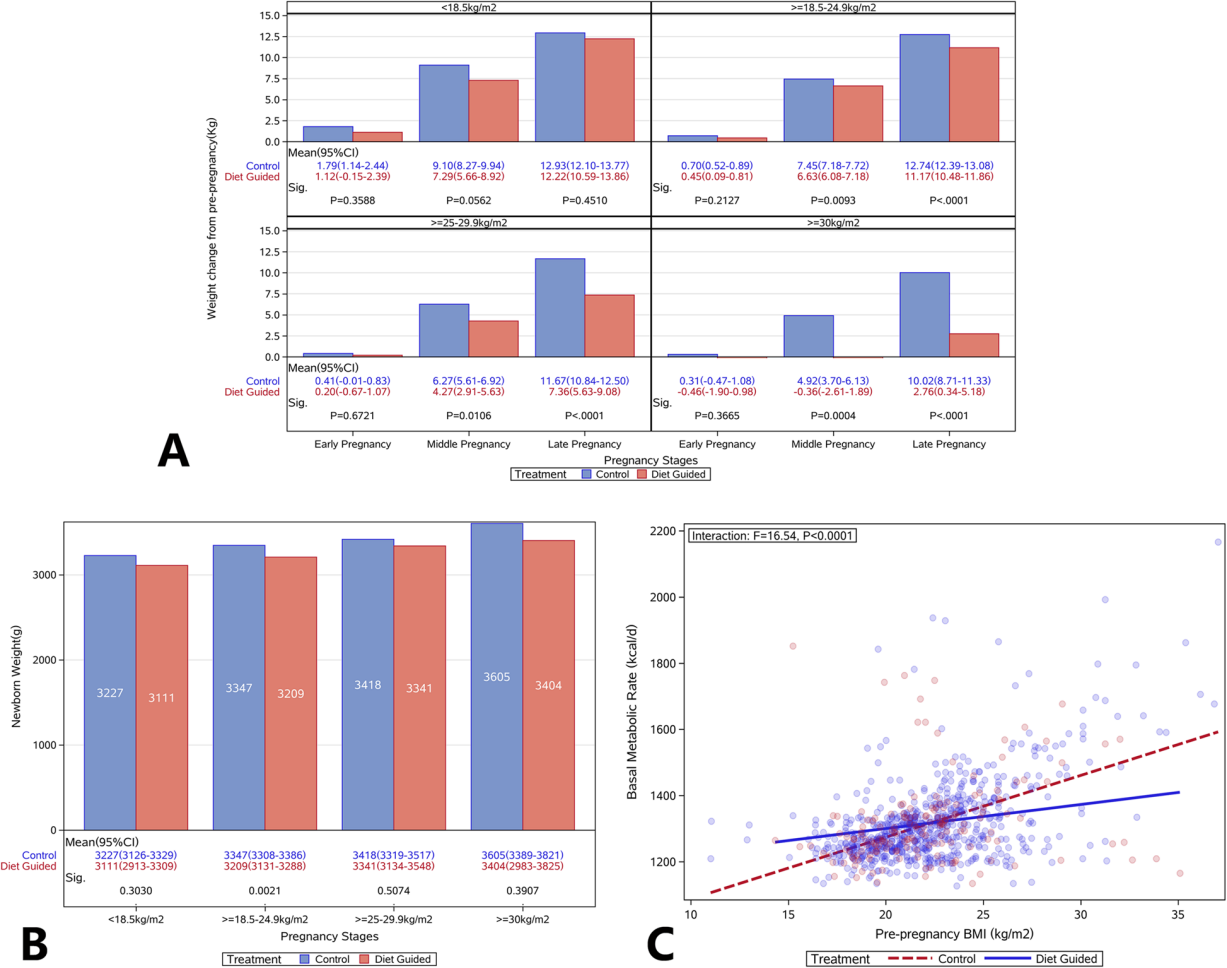
Comparison of gestational weight gain, birth weight, and basal metabolic rate in pregnant women with different pre-pregnancy body mass index. **A** Pregnant women were divided into four groups according to the pre-pregnancy BMI, and the GWG was analyzed in each stage of pregnancy; **B** The birth weight in pregnant women with different pre-pregnancy BMI; **C** The BMR of pregnant women with different pre-pregnancy BMI. Body Mass Index (BMI); gestational weight gain (GWG); basal metabolic rate (BMR)

After dietary intervention, the GWG decreased significantly in pregnant women with different pre-pregnant BMI, and the decrease of GWG was more evident in women with higher BMI (Fig. 4A), especially in the pre-pregnant obesity group. In the first trimester, the GWG was − 1.90-0.98 kg (− 0.26–0.15/week) kg; in the second trimester, the GWG was − 2.61 - 1.89 kg (− 0.27–0.31 kg/week); in the third trimester, the GWG was 0.34–5.18 kg (0.003–0.54 kg/week), which were significantly lower than the GWG in the control group and the recommended GWG in the guidelines. In several subjects with obesity, the weight loss was 1–6 kg during the pregnancy, the birth weight was 3110–4150 g, and there were no premature delivery and SGA.

Compared with the control group, a significant difference in the birth weight was only observed in the pre-pregnancy normal weight subjects after dietary guidance, suggesting that the influence of pre-pregnancy weight masked the effect of weight control during the pregnancy (Fig. 4B).

The daily energy intake was compared in the first trimester of women with different pre-pregnant BMI. Results showed the daily basal metabolic rate (BMR; kcal/d) of pregnant women increased significantly with the increase of BMI (Fig. 4C). After dietary and exercise guidance, the BMR increased in women with lower pre-pregnancy BMI, but decreased in women with higher pre-pregnancy BMI (*P* < 0.0001). This suggests that dietary and exercise guidance improves the BMR in pregnant women with higher or lower BMR.

**Fig. 5:**
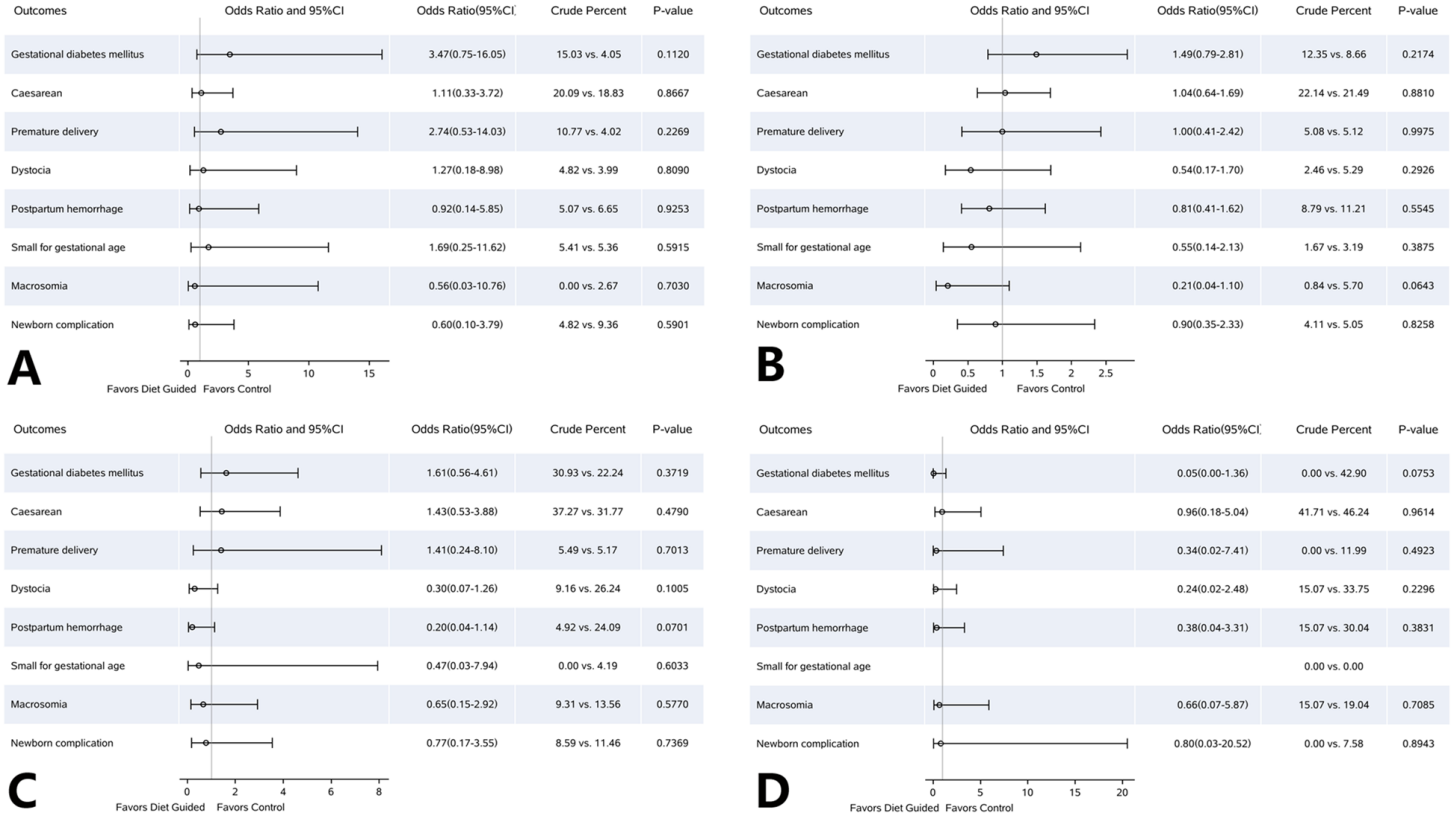
Comparison of perinatal outcomes of the women and infants in pregnant women with different pre-pregnancy body mass index. **A** underweight (BMI < 18.5 kg/m^2^) group; **B** normal weight (BMI = 18.5 ~ 25 kg/m^2^) group; **C** overweight (BMI = 25 ~ 30 kg/m^2^) group; **D** obesity (BMI ≥ 30 kg/m^2^) group. Body Mass Index (BMI)

Logistic regression analysis showed that, in the pre-pregnancy underweight women, the OR of clinical indicators was close to 1 between the DG group and the control group, suggesting no marked difference. In the pre-pregnancy normal weight, overweight, and obesity subgroups, the incidences of macrosomia, dystocia, postpartum hemorrhage, SGA, and neonatal complications tended to decrease after dietary guidance (OR < 1). In the women with different pre-pregnancy BMI, the incidences of macrosomia, postpartum hemorrhage, dystocia, cesarean section, and GDM increased significantly with the increase of pre-pregnancy BMI (Fig. 5).

## Discussion

In the present study, the singleton pregnant women in our hospital were retrospectively analyzed, the characteristics of GWG were explored in women with different pre-pregnancy BMI, and the effects of diet management during the pregnancy on the maternal and neonatal outcomes were further investigated. Our results showed that the dietary guidance and appropriate exercise were effective in reducing GWG as compared to the simple nutrition guidance, and even some women with pre-pregnancy obesity achieved weight loss during pregnancy. Compared with the control group, the GWG in the DG group reduced significantly in each stage of pregnancy, especially in the second and third trimesters. This indicates it is important to control body weight during the pregnancy. In general, the GWG was positively related to the birth weight. Of note, as compared to the control group, birth weight was lower in the DG group when the GWG was comparable during the pregnancy. It was surprising that the GWG was negatively related to the birth weight in the pre-pregnancy obesity subgroup. That is, after weight control, the birth weight was higher when the GWG was reduced, which suggests no relationship between GWG and birth weight. However, when the mean BMI was used, the pre-pregnancy BMI and GWG positively affected the birth weight. It suggests that the higher pre-pregnancy body weight masks the effect of weight control during pregnancy in the pre-pregnancy obese women.

Compared with the control group, the incidences of dystocia, postpartum hemorrhage, macrosomia, SGA, and neonatal complications significantly reduced in the DG group, and the incidence of preterm birth remained unchanged. The dietary and exercise guidance improved the BMR of pregnant women with higher or lower BMR. In addition, our results showed several women with pre-pregnancy obesity achieved weight loss during the pregnancy, but premature birth and SGA were not observed. This indicates that the active weight control during pregnancy in the pre-pregnancy obese women may not increase the risk of preterm birth and SGA, but has the possibility of reducing the risk of perinatal complications because pre-pregnancy weight has greater influence on the risk of complications during the pregnancy and perinatal period.

Our study showed that the incidences of macrosomia, postpartum hemorrhage, dystocia, cesarean section and GDM increased significantly with the increase of pre-pregnancy BMI, suggesting that pre-pregnancy weight contributes more to the outcomes such as macrosomia, GDM and way of delivery. This also highlights the importance of weight control before pregnancy. Some studies have shown that increased pre-pregnancy BMI is an independent risk factor for GDM because high pre-pregnancy BMI increases the adverse maternal and infant outcomes, and the incidence of GDM further increases in pregnant women with overweight or obesity [25–27]. The increased BMI in GDM pregnant women is associated with adverse pregnancy outcomes, and pregnant women with higher BMI are more likely to have higher birth weights and higher incidence of cesarean section [3]. A large international multi-ethnic study indicates that the pre-pregnancy obesity significantly increases the risk of preterm birth [28]. The greater the pre-pregnancy BMI, the higher the birth weight is, and the higher the incidence of macrosomia is [29]. The pre-pregnancy overweight and excessive GWG may increase the risk of overweight and obesity in preschool children, and the pre-pregnancy overweight has a greater influence as compared to GWG [5].

Therefore, both pre-pregnancy obesity and excessive GWG may increase the transfer of heat to the developing fetus and affect fetal development, which threatens the maternal and infant health and are related to the increased risk of miscarriage, GDM, stillbirth, premature delivery and cesarean section [30]. For example, pre-pregnancy obesity and excessive GWG can aggravate insulin resistance, cause blood glucose and lipid dysmetabolism during the pregnancy, and increase the risk of diseases (such as GDM) [2, 31, 32].

In addition to active weight control before pregnancy, reasonable pregnancy management is also needed to control the GWG, which may effectively reduce the birth weight and decrease maternal and infant complications during the perinatal period. A meta-analysis of clinical studies in 2022 has revealed that dietary and excise guidance seems to be an optimal way to prevent GDM and overweight during pregnancy [33]. The protocol for pregnancy management is clinically feasible in the present study. The weight control during pregnancy is mainly dependent on diet control and appropriate exercise. Our experience shows following programs are feasible: nutrition education via multiple ways, adjustment of dietary structure, administration of diet with low-glycemic load, reduction of caloric intake (daily energy intake is equivalent to about 150% of BMR), and appropriate exercise (10,000 steps/day in the second trimester, 20,000 steps/day in the third trimester).

The American College of Obstetrics and Gynecology and the Physical Activity Guidelines for Americans in 2018 recommend ≥30–150 min of aerobic exercise per week [34, 35]. There is evidence showing that the incidences of adverse outcomes (such as GDM, gestational hypertension, and preeclampsia) reduce by 38–41% in women receiving excise guidance during the pregnancy as compared to the control group, which is accompanied by a reduction of GWG [36]. Another randomized clinical trial investigated the influence of exercise initiated early in pregnancy and performed at least 30 min (3 times per week) on the GDM. Their results showed cycling exercise significantly reduced the frequency of GDM in overweight/obese pregnant women. This effect was ascribed to that exercise initiated early in pregnancy decreased the GWG before the mid-second trimester. However, exercise failed to increase the risk of preterm birth or reduce the mean gestational age at birth in their study [27].

In a randomized, parallel-group, controlled trial, the influence of telehealth lifestyle intervention on the GWG was investigated in women with overweight or obesity. The results showed telehealth lifestyle intervention could improve the healthy behaviors and insulin resistance and reduce the GWG in these women [37]. In the present study, a social software was employed for the lifestyle intervention, and results were consistent with above mentioned.

Barone et al. reported that higher sedentary behavior and lower standing, stepping, and steps per day trajectories in the second and third trimesters increased the risks of adverse pregnancy outcomes [20]. Andersen et al. employed an accelerometer to assess physical activity during pregnancy. Their results showed inverse linear relationships of pre-pregnancy BMI with the mean number of steps per day and the mean metabolic equivalent of tasks per day. This means that pre-pregnancy overweight or obesity may reduce physical activity during pregnancy, increasing the risk of sedentary behavior during pregnancy [21]. Therefore, for women with pre-pregnancy obesity, increasing the physical activity during pregnancy is important to reduce the GWG. Therefore, it is recommended that weight control and weight management during the pregnancy are needed for women with high pre-pregnancy BMI, which may reduce the risks of maternal and infant complications related to obesity. Our results showed that the second and third trimesters were the best periods for weight control, and interventions to weight control in the second and third trimesters are more likely to prevent fat gain, which is consistent with previously reported [38].

## Conclusions

In summary, for pregnant women with higher pre-pregnancy BMI, strict weight control is required to reduce the maternal and neonatal complications related to obesity. Compared with GWG, the pre-pregnancy BMI has greater influence on the birth weight and the maternal and infant complications, and may even compromise the benefits of weight control during pregnancy.

Therefore, the control of body weight before pregnancy is of great importance. For women with high pre-pregnancy BMI, weight control program (nutrition education via multiple ways, adjustment of dietary compositions, administration of diet with a low-glycemic index, reduction of calorie intake and appropriate exercise, etc.) is recommended to reduce GWG or even reducing weight during the pregnancy, which may reduce the birth weight, decrease the incidences of dystocia and postpartum hemorrhage without increasing maternal and infant complications, and tend to reduce the incidences of premature delivery and SGA.

## Data Availability

The datasets used and analyzed during the current study are available from the corresponding author upon reasonable request.

## Abbreviations

GWG: Gestational weight gain
GDM: Gestational diabetes mellitus
BMI: Body mass index
DG: Diet guidance
SGA: Small for gestational age
NICU: Neonatal intensive care unit
ATE: Average treatment effect
LS: Least Square
OR: Odds Ratio
BMR: Basal metabolic rate

## References

[1] Afshin A, Reitsma MB, Murray CJL (2017). Health effects of overweight and obesity in 195 countries. N Engl J Med.

[2] Yen IW, Lee CN, Lin MW, Fan KC, Wei JN, Chen KY (2019). Overweight and obesity are associated with clustering of metabolic risk factors in early pregnancy and the risk of GDM. PLoS One.

[3] Ganer Herman H, Dekalo A, Jubran L, Schreiber L, Bar J, Kovo M (2019). Obstetric outcomes and placental findings in gestational diabetes patients according to maternal prepregnancy weight and weight gain. J Matern Fetal Neonatal Med.

[4] Catalano PM, Shankar K (2017). Obesity and pregnancy: mechanisms of short term and long term adverse consequences for mother and child. BMJ..

[5] Adane AA, Tooth LR, Mishra GD (2019). The role of offspring's birthweight on the association between pre-pregnancy obesity and offspring's childhood anthropometrics: a mediation analysis. J Dev Orig Health Dis.

[6] Samuelsson AM, Matthews PA, Argenton M, Christie MR, McConnell JM, Jansen EH (2008). Diet-induced obesity in female mice leads to offspring hyperphagia, adiposity, hypertension, and insulin resistance: a novel murine model of developmental programming. Hypertension..

[7] Zhou L, Xiao X (2018). The role of gut microbiota in the effects of maternal obesity during pregnancy on offspring metabolism. Biosci Rep.

[8] Hsu MH, Chen YC, Sheen JM, Huang LT (2020). Maternal obesity programs offspring development and resveratrol potentially reprograms the effects of maternal obesity. Int J Environ Res Public Health.

[9] Álvarez-Bueno C, Cavero-Redondo I, Lucas-de la Cruz L, Notario-Pacheco B, Martínez-Vizcaíno V (2017). Association between pre-pregnancy overweight and obesity and children's neurocognitive development: a systematic review and meta-analysis of observational studies. Int J Epidemiol.

[10] Shrestha N, Ezechukwu HC, Holland OJ, Hryciw DH (2020). Developmental programming of peripheral diseases in offspring exposed to maternal obesity during pregnancy. Am J Physiol Regul Integr Comp Physiol.

[11] Dearden L, Buller S, Furigo IC, Fernandez-Twinn DS, Ozanne SE (2020). Maternal obesity causes fetal hypothalamic insulin resistance and disrupts development of hypothalamic feeding pathways. Mol Metab.

[12] Dong M, Zheng Q, Ford SP, Nathanielsz PW, Ren J (2013). Maternal obesity, lipotoxicity and cardiovascular diseases in offspring. J Mol Cell Cardiol.

[13] Gaillard R (2015). Maternal obesity during pregnancy and cardiovascular development and disease in the offspring. Eur J Epidemiol.

[14] Alfaradhi MZ, Fernandez-Twinn DS, Martin-Gronert MS, Musial B, Fowden A, Ozanne SE (2014). Oxidative stress and altered lipid homeostasis in the programming of offspring fatty liver by maternal obesity. Am J Physiol Regul Integr Comp Physiol.

[15] Glastras SJ, Tsang M, Teh R, Chen H, McGrath RT, Zaky AA (2016). Maternal obesity promotes diabetic nephropathy in rodent offspring. Sci Rep.

[16] Edlow AG (2017). Maternal obesity and neurodevelopmental and psychiatric disorders in offspring. Prenat Diagn.

[17] Myer ENB, Roem JL, Lovejoy DA, Abernethy MG, Blomquist JL, Handa VL (2018). Longitudinal changes in pelvic floor muscle strength among parous women. Am J Obstet Gynecol.

[18] Shieh C, Cullen DL, Pike C, Pressler SJ (2018). Intervention strategies for preventing excessive gestational weight gain: systematic review and meta-analysis. Obes Rev.

[19] Institute of Medicine NRC (2009). Weight gain during pregnancy: reexamining the guidelines.

[20] Barone Gibbs B, Jones MA, Jakicic JM, Jeyabalan A, Whitaker KM, Catov JM (2021). Objectively measured sedentary behavior and physical activity across 3 trimesters of pregnancy: the monitoring movement and health study. J Phys Act Health.

[21] Andersen MB, Ostenfeld EB, Fuglsang J, Møller M, Daugaard M, Ovesen PG (2020). Maternal prepregnancy body mass index and physical activity during pregnancy assessed by accelerometer. Am J Obstet Gynecol MFM.

[22] Committee on Practice Bulletins-Obstetrics. Practice Bulletin No. 183: Postpartum Hemorrhage. Obstet Gynecol. 2017;130:e168–e86. 10.1097/AOG.0000000000002351 28937571

[23] Bullough S, Navaratnam K, Sharp A (2021). Investigation and management of the small for gestational age fetus. Obstet Gynaecol Reprod Med.

[24] Cunningham JJ (1980). A reanalysis of the factors influencing basal metabolic rate in normal adults. Am J Clin Nutr.

[25] Leng J, Li W, Zhang S, Liu H, Wang L, Liu G (2015). GDM Women’s pre-pregnancy overweight/obesity and gestational weight gain on offspring overweight status. PLoS One.

[26] McIntyre HD, Catalano P, Zhang C, Desoye G, Mathiesen ER, Damm P (2019). Gestational diabetes mellitus. Nat Rev Dis Primers.

[27] Wang C, Wei Y, Zhang X, Zhang Y, Xu Q, Sun Y (2017). A randomized clinical trial of exercise during pregnancy to prevent gestational diabetes mellitus and improve pregnancy outcome in overweight and obese pregnant women. Am J Obstet Gynecol.

[28] Liu B, Xu G, Sun Y, Du Y, Gao R, Snetselaar LG (2019). Association between maternal pre-pregnancy obesity and preterm birth according to maternal age and race or ethnicity: a population-based study. Lancet Diabetes Endocrinol.

[29] Patro Golab B, Santos S, Voerman E, Lawlor DA, Jaddoe VWV, Gaillard R (2018). Influence of maternal obesity on the association between common pregnancy complications and risk of childhood obesity: an individual participant data meta-analysis. Lancet Child Adolesc Health.

[30] Galtier-Dereure F, Boegner C, Bringer J (2000). Obesity and pregnancy: complications and cost. Am J Clin Nutr.

[31] Marchi J, Berg M, Dencker A, Olander EK, Begley C (2015). Risks associated with obesity in pregnancy, for the mother and baby: a systematic review of reviews. Obes Rev.

[32] O'Malley EG, Reynolds CME, Killalea A, O'Kelly R, Sheehan SR, Turner MJ (2020). Maternal obesity and dyslipidemia associated with gestational diabetes mellitus (GDM). Eur J Obstet Gynecol Reprod Biol.

[33] Wu S, Jin J, Hu KL, Wu Y, Zhang D (2022). Prevention of gestational diabetes mellitus and gestational weight gain restriction in overweight/obese pregnant women: a systematic review and network Meta-analysis. Nutrients..

[34] ACOG Committee Opinion No. 650: physical activity and exercise during pregnancy and the postpartum period. Obstet Gynecol. 2015;126:e135–e42. 10.1097/AOG.0000000000001214 26595585

[35] Piercy KL, Troiano RP, Ballard RM, Carlson SA, Fulton JE, Galuska DA (2018). The physical activity guidelines for Americans. JAMA..

[36] Davenport MH, Ruchat SM, Poitras VJ, Jaramillo Garcia A, Gray CE, Barrowman N (2018). Prenatal exercise for the prevention of gestational diabetes mellitus and hypertensive disorders of pregnancy: a systematic review and meta-analysis. Br J Sports Med.

[37] Ferrara A, Hedderson MM, Brown SD, Ehrlich SF, Tsai AL, Feng J (2020). A telehealth lifestyle intervention to reduce excess gestational weight gain in pregnant women with overweight or obesity (GLOW): a randomised, parallel-group, controlled trial. Lancet Diabetes Endocrinol.

[38] Most J, Altazan AD, Hsia DS, Beyl RA, Redman LM (2020). Body composition during pregnancy differs by obesity class. Obesity (Silver Spring).

